# Urban water supply systems’ resilience under earthquake scenario

**DOI:** 10.1038/s41598-022-23126-8

**Published:** 2022-11-29

**Authors:** Mo’Tamad H. Bata, Rupp Carriveau, David S.-K. Ting

**Affiliations:** Turbulence and Energy Lab, Ed Lumley Centre for Engineering Innovation, University of Windsor, Windsor, ON Canada

**Keywords:** Civil engineering, Natural hazards

## Abstract

Threats to water supply systems have increased in number and intensity. Natural disasters such as earthquakes have caused different types of damage to water distribution networks (WDN), particularly for those with aged infrastructure. This paper investigates the resilience of an existing water distribution network under seismic hazard. An earthquake generation model coupled with a probabilistic flow-based pressure driven demand hydraulic model is investigated and applied to an existing WDN. A total of 27 earthquake scenarios and 2 repair strategies were simulated. The analysis examined hydraulic resilience metrics such as pressure, leak demand, water serviceability, and population impacted. The results show that nodal pressure drops below nominal pressure and reaches zero in some earthquake scenarios. Leak demand could reach to more than 10 m^3^/s within hours following an earthquake. Water serviceability drops to a low of 40% and population impacted reaches up to 90% for a 6.5 M earthquake, for example. This study highlights and quantifies vulnerabilities within the simulated WDN. The tools outlined here illustrate an approach that can: (1) ultimately help to better inform utility water safety plans, and (2) prepare proactive strategies to mitigate/repair before a hazard of this nature occurs.

## Introduction

Water Distribution Networks (WDNs) are Critical Infrastructure (CI)^1^. WDN consist of systems, facilities, technologies, services, etc. essential to the well-being of people and sustainable economy. WDNs face a wide range of threats capable of causing significant damage and service disruptions. These threats include aging infrastructure, natural disasters, and man-made hazards. WDNs are similar in their layout and functions but differ in their size, location, and vulnerabilities. Accordingly, water utilities are implementing strategies and developing tailored Water Safety Plans (WSP) to increase their WDN resilience and overcome such threats.

Natural disasters like earthquakes, droughts, fires, hurricanes, floods, and heavy storms have caused a spectrum damage to WDNs, especially for those with aged infrastructure. Researchers have proposed methods to estimate the aftermath of natural disasters on critical infrastructure like water supply systems. Previous studies reported damages that include pipe leaks and breaks; failure of treatment plants, pumps, reservoir, and tanks; power outages; losses of water quality, reduced access to supplies and facilities; and other negative impacts on systems personnel^2–4^. After studying the damage a natural disaster can inflict on water supply systems, The Pan-American Health Organization (PAHO)^5^ reported that earthquakes have the most destructive potential to WDN. That is because: (1) earthquakes cause cascading damages represented by fires, power outages, and structural destruction to WDN assets and facilities, and (2) WDN components are buried underground where the seismic attenuation is more intense, and where damage is not easily detected and repaired^6,7^.

For example, the massive San Francisco earthquake that lasted one minute in 1906^8^ destroyed thousands of pipes, caused shortage in fire flow resulting in a fire that lasted three days, killed 800 people, and left massive property damage. The 1995 Kobe earthquake^9^ damaged more than 4000 pipes and caused loss of water service for more than one million people. The San Fernando and the Northridge earthquakes^10^ damaged tens of water mains and water service was lost for weeks. More recent earthquakes have resulted in significant damages to WDN despite the advancements in anti-seismic materials and technologies. The South Napa earthquake in 2014 further demonstrated the vulnerability of WDNs. More than 150 water main breaks were reported which left firefighters with an insufficient quantity of water to fight six major post-earthquake fires^11^. Carter^12^ stressed the concern of mold growth due to water pipe failure causing more damage than the shaking itself. The Kumamoto earthquake in 2016 is another recent reminder of WDN vulnerability to seismic threats. The earthquake intermittently cut off the water supply for several days^13^.

The reliability and resilience of water supply systems under seismic action has been studied over the past few decades. To study this topic, researchers applied and proposed different methods such as deterministic and probabilistic seismic hazard analyses; analytical models (e.g., numerical models of buried pipelines, models of pipelines deterioration, component interdependency models, and flow-based models^14^. A highlight summary of these studies is presented in Table 1.

**Table 1:** A summary of previous studies on the water supply systems under seismic hazard.

Reference	Description
Shinozuka et al.^15^. Serviceability of water transmission systems under seismic hazard	Used Monte-Carlo Simulation to evaluate the seismic reliability of the Los Angeles WDN
O’Rourke et al.^16^. Seismic response of buried pipes	Simulated a damaged WDN under seismic hazard and measured its serviceability
Markov et al.^17^. An evaluation of seismic serviceability of water supply networks with application to San Francisco auxiliary water supply system	Developed and applied an algorithm for the hydraulic analysis of a seismically damaged WDN. System service ratio was also measured in this study
Hwang et al.^18^. Seismic performance assessment of water delivery systems	Evaluated the WDN service ratio under seismic hazard in Memphis, Tennessee. The evaluation was carried through hydraulic simulation
SelÇuk and Yücemen^19^. Reliability of lifeline networks with multiple sources under seismic hazard	Evaluated multiple source WDN reliability under seismic hazard through a probabilistic model using LIFPACK software
Shi and O’Rourke^20^. Seismic response modeling of water supply systems	Proposed a comprehensive model for the seismic hazard evaluation of WDN
Wang and O’Rourke^21^. Seismic performance evaluation of water supply systems	Assessed the safety of five WDN in Los Angeles under seismic hazard
Dueñas‐Osorio et al.^22^. Seismic response of critical interdependent networks	Studied the interdependency between WDN and power networks under seismic hazard
Adachi and Ellingwood^23^. Serviceability of earthquake-damaged water systems: effects of electrical power availability and power backup systems on system vulnerability	Investigated the level of damaged WDN serviceability under earthquake hazard, and evaluated the system vulnerability in the presence/absence of electrical power backup
Bonneau and O’Rourke^24^. Water supply performance during earthquakes and extreme events	Proposed an improved hydraulic model to evaluate WDN stability under an extreme event like earthquake hazard
Fragiadakis and Christodoulou^25^. Seismic reliability assessment of urban water networks	Used the pipeline survival function to perform seismic risk assessment of WDN
Hou and Du^26^. Comparative Study on Hydraulic Simulation of Earthquake-Damaged Water Distribution System	Quantified seismic damage on WDN through hydraulic analysis
Yoo et al.^7^. Seismic Hazard Assessment Model for Urban Water Supply Networks	Proposed reliability evaluation model for seismic hazard for water supply network (REVAS.NET) and applied it on WDN in South Korea
Makhoul et al.^27^. Earthquake damage estimations of Byblos potable water network	Applied a probabilistic approach to estimate the earthquake damage on WDN buried pipelines
Salgado-Gálvez et al.^28^. Probabilistic assessment of annual repair rates in pipelines and of direct economic losses in water and sewage networks: application to Manizales, Colombia	Applied a probabilistic approach to estimate the earthquake damage on WDN and sewage buried pipelines
Yoon et al.^29^. A comprehensive framework for seismic risk assessment of urban water transmission networks	Presented a comprehensive assessment of WDN components under seismic hazard
Yoo et al.^30^. Comparative Study of Hydraulic Simulation Techniques for Water Supply Networks under Earthquake Hazard	Compared different hydraulic simulation techniques for WDN under seismic hazard
Yoon et al.^14^. A comprehensive approach to flow-based seismic risk analysis of water transmission network	Applied a flow-based approach to assess the WDN performance under seismic hazard
Paez et al.^31^. Battle of Post disaster Response and Restoration	Investigated response and restoration of water service scenarios after the occurrence of five earthquake scenarios that cause structural damage in a water distribution system
Uma et al.^32^. Planning for resilience of water networks under earthquake hazard: A case study for Rotorua District, New Zealand	Presented an approach to assess risks and damages to water supply systems under earthquake hazard

A review of these previous studies reveals that most lack a realistic hydraulic simulation coupled with an earthquake generation model. Few studies applied pressure driven demand analysis (PDD) when simulating a simultaneously disruptive earthquake, or when PDD is adopted, probabilistic simulations were not considered. Simulations that applied demand driven analysis (DDA) assumed that nodal demand is met regardless of pressure gradient. This is rarely the case during a disruptive event like an earthquake. Other presented simulations applied PDD without considering the wide range of possible simulations (i.e., probabilistic simulation), or applied on an imaginary WDN. This affects the reliability of the analyses significantly. In this study, an earthquake generation model is coupled with a probabilistic flow-based PDD hydraulic model. The coupling is developed and applied to an existing WDN in M-City in Ontario, Canada. While this is a case study, the overarching findings and implications may be used to improve predictive water hazard models in general.

## Methodology

**Figure 1:**
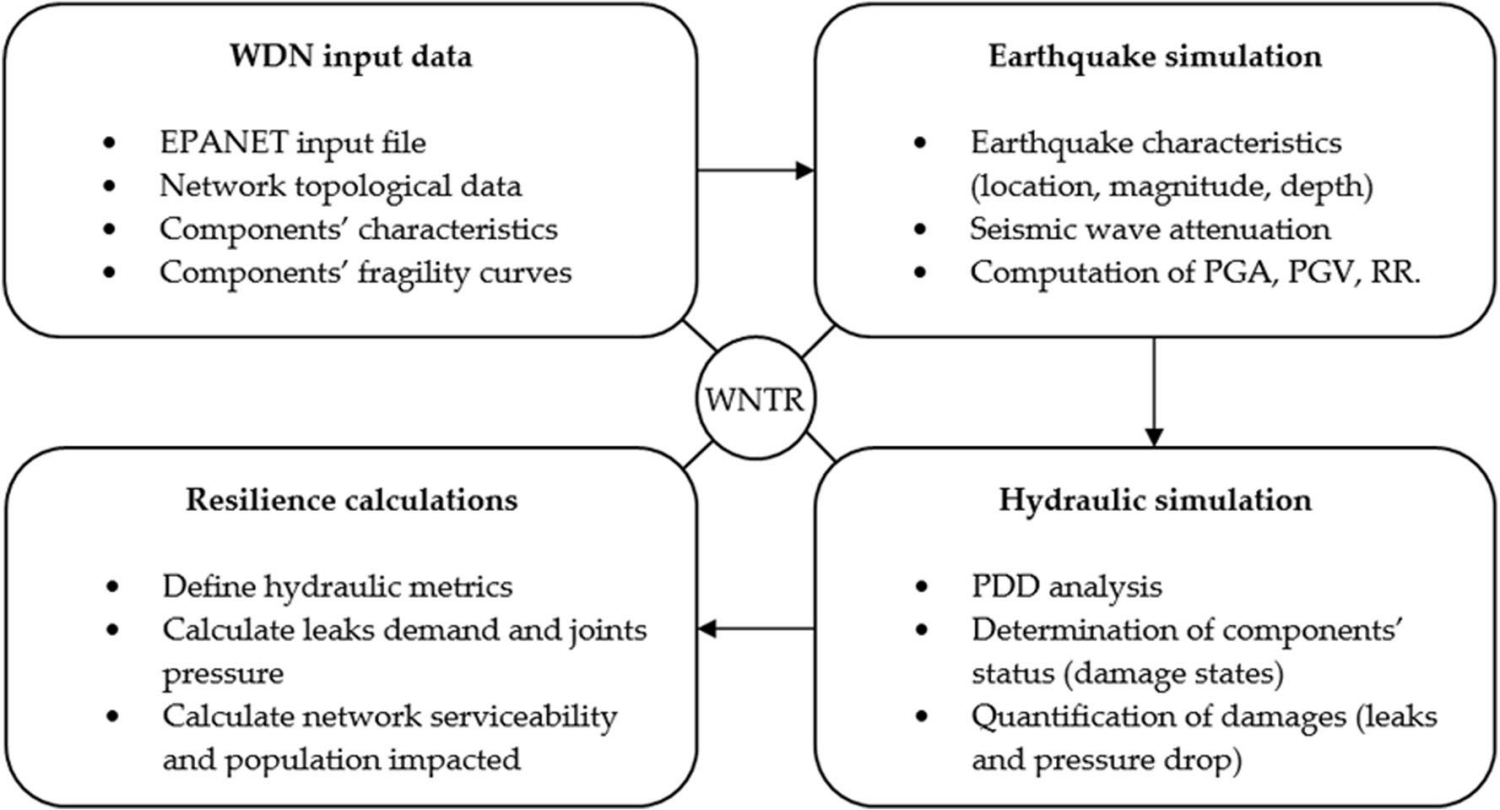
Flowchart of earthquake simulation in WDN.

This section describes the methods engaged to simulate earthquake hazards on this specific WDN. Figure 1 depicts the four sub-modules built for this task in the water network tool for resilience (WNTR)^33^. In sub-module 1, an input file (i.e., has the .INP extension) that contains descriptive data of the WDN is compiled in EPANET^34^. In this file, system components and their characteristics are entered. For example, size, location, elevation, age, building materials, inlet and outlet type data are entered to describe storage tanks. For pipes, typical data includes location, slope, diameter, upstream and downstream joints, material, etc. Then, system components are assigned fragility curves as described in “WDN components failure” section.

Sub-module 2, house the earthquake simulation process. First, a pre-determined hypothetical scenario is cast where an earthquake location, magnitude, and depth are assigned. The values for these characteristics could be chosen randomly or, as in this study, based on knowledge of the system. For example, to predict a worst-case scenario, a highly probable magnitude, a particularly sensitive location (e.g., near pumping station), and a wider depth are selected. Second, proper Ground Motion Prediction Equations (GMPE) are assigned to represent the seismic wave attenuation. Location, topography, and simulated earthquake characteristics are the main considerations in this step. Third, a formulation of peak ground acceleration (PGA), peak ground velocity (PGV), and repair rate (RR) are inserted and computed for each scenario.

In the third sub-module, simulation of the WDN hydraulics is performed using the PDD approach (“Hydraulic analysis” section). Then, using fragility curves and the computed values of PGA, PGV, and RR, the status of each WDN component is determined. The last step of this sub-module is to quantify system damages. For example, if the shaking had caused a power outage in one pump and multiple leaks in tanks, joints, and pipes; subsequent pressure drops, and leak demand amounts, and locations for this damage are quantified in this step.

WDN responsivity and serviceability under earthquake hazard is investigated through resilience metrics in the fourth sub-module (“Network resilience” section). In this sub-module, resilience metrics are defined, formulated, and calculated. This sub-module may be considered as a summary of the simulation results which is very important for analysts and decision-makers.

As this simulation is a scenario with multiple outcomes, Monte Carlo simulation is used to tackle the uncertainty associated with multiple realizations and to estimate the probabilistic seismic reliability. For meaningful results and as a validation of the simulation, it is recommended to consider a large number of iterations^7^. In this work, fifty realizations of each earthquake scenario (i.e., magnitude, location, and depth) are considered. The number of realizations was adopted from Klise et al.^4^ and can vary depending on the simulating machine capacity and speed.

### Earthquake occurrence

The effects of an earthquake on a WDN vary depending on earthquake intensity, location, and depth; topological characteristics; and WDN layout and facilities. When constructing an earthquake generation module, it is crucial to choose the input earthquake characteristics based on these factors^14^. Indubitably, the input earthquake will differ for each study area. According to Hazards United States Multi-Hazard (HAZUS-MH), six different options could be used to define an earthquake module; some of which are customized for the United States data^35^. Table 2 presents these options.

**Table 2:** Options for defining an earthquake module.

Option	Description
Historical epicentre	Scenarios are based on historical events
Source event	Scenarios are based on seismic event from the source event database
Arbitrary event	Scenarios are based on fault type, event type, epicentre, magnitude, depth, width, and fault rupture characteristics, with the use of an applicable attenuation function
Probabilistic hazard	Scenarios are based on probable return period and magnitude or annualized loss
User-supplied hazard	Scenarios are based on ground motion data supplied by the user
USGS ShakeMap	Scenario based on a USGS ShakeMap extensible markup language (XML) grid file for a recent, historic or scenario event

In this paper, the input earthquake events are extracted from historical data of the target WDN and expanded to arbitrary events. Historical data show that for a radius of 100 km, M-City has experienced earthquakes in the past four centuries with a maximum intensity of 4.1 M and a maximum depth of 18 km^36^. Historical data also shows that about 70%, 20%, 5%, and 5% of earthquakes had an intensity between 2 and 3 M, less than 2 M, between 3 and 4 M, and more than 4 M, respectively. Historically, WDN in the study area have not yet experienced devastating damages to their components caused by seismic hazards. However, the occurrence of a higher intensity earthquake will most likely create a new case of risks and damages to the WDN in the region.

### Earthquake attenuation law

Earthquakes Canada defines an earthquake as “The sudden release of stored elastic energy caused by the sudden fracture and movement of rocks along a fault”^36^. Some of the energy is released in the form of seismic waves, that cause the ground to shake. This ground shaking causes several ground motions depending on the transmitted energy path and geological characteristics of earth surface^37^. Ground Motion Prediction Equation (GMPE) formulates this process of releasing energy from the earthquake epicentre to the earth’s surface. Two physical representations of this motion are velocity and acceleration of the seismic waves. When investigating the risks of seismic hazard, one should consider the peak of such motions (i.e., PGV and PGA). According to HAZUS-MH, vulnerabilities of buried pipes are related to PGV, and vulnerabilities of other WDN components (e.g., tanks, pumps, and water treatment plants) are related to PGA^38^. Attenuation models are developed for a certain earthquake and a particular region. Therefore, modelers usually use the average of several empirical models to conclude a general behaviour^4,7,39^. In this study, the GMPE, Eq. 1, for the PGV in a soil characteristic topology proposed by Kawashima et al.^40^; and the GMPE, Eq. 2, for the PGA proposed by Yu and Jin^41^ are adopted.

1 $$PGV = 10^{{ - 0.285 + 0.711M - 1.85\left( {R + 17} \right)}}$$

2 $$PGA = 403.8\times 10^{0.265M} \left( {R + 30} \right)^{ - 1.218}$$

where M is the unitless earthquake magnitude, and R is the earthquake depth measured from the epicentre in kilometers (km). PGV is frequently used to estimate pipes repair rate (RR) which is defined as the number of repairs needed per one kilometer length of pipe (repairs/km). Equations 3 and 4 represent linear and power law RR models from American Lifelines Alliance^39^, respectively. Attenuation models are developed for a specific location and earthquake. Therefore, correction factors are added to account for soil and pipe characteristics^42^. Table 3 and Eq. 5 display the correction factor and their categories and weights adopted from Isoyama et al.^42^.

3 $$RR = 0.00187\, \times \,PGV$$

4 $$RR = 0.00108\, \times \,PGV^{1.173}$$

5 $$C = C_{t} \, \times \,C_{d} { }\, \times \,{ }C_{l} { }\, \times \,C_{m}$$

**Table 3:** Correction factors used to modify repair rate (RR).

**Topography (C**_**t**_**)**					
Category	Stiff alluvial	Alluvial	Disturbed hill	Terrace	Narrow valley
Factor	0.4	1.0	1.1	1.5	3.2
**Pipe diameter (C**_**d**_**)**					
Category	Large	Medium	Small	Very small	
Factor	0.5	0.8	1.0	1.6	
**Liquification potential (C**_**l**_**)**					
Category	None	Partial	Total		
Factor	1.0	2.0	2.4		
**Pipe material (C**_**m**_**)**					
Category	DCIP	SP	HI-3P	PE	
Factor	0.3	0.3	0.8	0.8	
Category	CIP	PVC	PV	ACP	
Factor	1.0	1.0	1.0	1.2	

DCIP, ductile cast iron pipe; SP, steel pipe; HI-3P, high impact 3-layer pipe; PE, polyethylene pipe; CIP, cast iron pipe; PVC, polyvinyl chloride pipe; PV, polyvinyl pipe; ACP, asbestos cement pipe.

### WDN components failure

Post-earthquake damages to the WDN are caused by ground motion. Fragility curves expressed in terms of ground motion functions are a common method used to predict damages to a WDN. Here, Fragility curves are statistical tools that predict the probability of a component reaching or exceeding a certain damage state under seismic excitation. WDN components such as tanks, pumps, and pipes can have different damage states. For example, pipe damage can be expressed in four states: breakage, major leak, minor leak, and no damage at all. A large database of earthquake characteristics and their damages to WDN components can be used to construct a type of fragility curve known as an empirical fragility curve. An example of this type of fragility curves is those reported in The American Lifelines Alliance reports^39,43^. In these reports, PGV and RR are often used to estimate damages to buried pipes, where PGA is used in the case of pumps and tanks.

### Hydraulic analysis

In this study, WDN analyses are carried out in two compatible software environments, EPANET and Water Network Tool for Resilience (WNTR). EPANET has high reliability in hydraulic analysis of a steady-state WDN, and an approachable user interface. WNTR has a spectrum of capabilities that align well with this work: the ability to add disruptive incidents and response strategies, performing probabilistic simulations, and computing resilience metrics were essential to this study.

Throughout the WDN, hydraulic analyses are carried out for all nodes and links. Every node and link in the water network has a mass balance equation that quantifies inflow, outflow, and possible storage. Mass balance equations adopted in EPANET are shown in Eq. 6^34^.

6 $$\mathop \sum \limits_{{p \in P_{n} }} q_{p,n} - { }D_{n}^{act} = 0\quad\, n{ } \in N{ }$$

where P_n_ is the set of pipes connected to node n, q_p,n_ is water flow rate (m^3^/s) from pipe p into node n, D^act^_n_ is the actual water demand leaving node n (m^3^/s), and N is the set of all nodes. Here, q_p,n_ is positive unless water is outflowing node n into pipe p.

To account for head losses in links, conservation of energy formulae are used. In our hydraulic analyses, the Hazen-Williams head loss formula, Eq. 7^34^, is selected in EPANET.

7 $$H_{nj} - { }H_{ni} { } = { }h_{l} = 10.667C^{ - 1.852} { }d^{ - 4.871} { }Lq^{1.852}$$

where h_L_ is the head loss in the pipe (m), C in the Hazen-Williams roughness coefficient (unitless), d is the pipe diameter (m), L is the pipe length (m), q is the water flow rate (m^3^/s), H_nj_ and H_ni_ are are the heads at the starting and ending nodes (m), respectively.

During a disruptive incident like an earthquake, where pressure gradients in the WDN are unsteady, applying PDD methods in hydraulic simulations are more realistic and accurate than DDA. In WNTR, the PDD that is used in hydraulic simulations is that proposed by Wagner et al.^44^. A formulation of this model is presented in Eq. 8.

8 $$D = { }\left[ {\begin{array}{*{20}c} { 0\quad p \le P_{0} } \\ {D_{f} \sqrt {\frac{{p{ } - { }P_{0} }}{{P_{f} - { }P_{0} }}{ }} \quad P_{0} < p < P_{f} } \\ {D_{f}\quad p \ge P_{f} } \\ \end{array} } \right]$$

where D is the actual demand delivered (m^3^/s), p is the pressure (Pa), P_0_ is the pressure below which the customers cannot receive any water (Pa), D_f_ is the expected demand (m^3^/s), and P_f_ is the pressure above which the expected demand should be received (Pa).

Another model that is crucial during a disruptive event is the leak model. Leaks are expected to occur in tanks, fittings, junctions, and pipes. Leaks negatively affect the hydraulics and serviceability of the WDN. In WNTR, leaks or leak demand can be modeled, and lost water can be quantified between the start of the disruptive event and the time of repair for those affected components. In cases where leaks are major, a breakage can be modelled by splitting a pipe into two sections and adding two new disconnected junctions at both ends. The equation, expressed here in general form as Eq. 9^45^ is used in WNTR to quantify leaks as a mass flowrate.

9 $$d^{leak} = C_{d} A \sqrt {2\rho P^{\alpha } }$$

where d^leak^ is leak demand (m^3^/s), C_d_ is the discharge coefficient, A is leak hole surface area (m^2^), ρ is the water density (kg/m^3^), P is the in-pipe gauge pressure (Pa), and α is a correction factor. Assuming a turbulent flow and large leaks out of pipes, values for C_d_ and α are set to 0.75 and 0.5, respectively^46^.

### Network resilience

In engineering, resilience of a system is defined as the time required to restore an equilibrium state after a disruptive event occurs^47^. The WDN resilience cycle begins in the design stage, then, is shaped through operations and maintenance, and finally tested through repair and mitigation processes. Each stage aims to reduce the effect of disruptive events when they occur. Quantifying WDN resilience is crucial to predict the possible system response to a variety of disruptive events. The United States Environmental Protection Agency (USEPA) classifies WDN resilience metrics into five categories: economic, hydraulic, topographic, water quality, and water security^48^. Selecting the most representative metric is important and depends on the disruptive event scenario, and available input data. In this study, hydraulic metrics are employed to measure the WDN resilience. This includes, pressure, demand, water serviceability, and population impacted. Earthquake expected damages to the WDN (e.g., leaks, breakages, power loss) and available hydraulic model data were the reason hydraulic metrics were chosen. Equations 10 and 11 compute water serviceability and population impacted, respectively.

10 $$WSA_{t} = \left( {\mathop \sum \limits_{n = 1}^{n = N} V_{nt} } \right)/\left( {\mathop \sum \limits_{n = 1}^{n = N} \tilde{V}_{nt} } \right)$$

11 $$Pl_{t} = \mathop \sum \limits_{n = 1}^{n = N} pop_{n} *d_{nt}$$

12 $$pop_{n} = q_{n} /R$$

13 $$d_{nt} = { }\left\{ {\begin{array}{*{20}l} 1 \hfill & { if\,\, V_{nt} /\tilde{V}_{nt} < threshold} \hfill \\ 0 \hfill &\quad\quad { otherwise} \hfill \\ \end{array} } \right\}$$

where WSA_t_ is the water serviceability of the WDN at time t, n is the node number of the total N network nodes, V_nt_ and Ṽ_nt_ are the actual and expected water volume received at node n at time t. For example, if we have a simple network of four nodes (n_1_, n_2_, n_3_, and n_4_) that at a time t have an expected demand of (Ṽ_1t_ = Ṽ_2t_ = Ṽ_3t_ = Ṽ_4t_ = 0.1 m^3^), and an actual demand of (V_1t_ = 0.06 m^3^, V_2t_ = 0.09 m^3^, V_3t_ = 0.09 m^3^, and V_4t_ = 0.08 m^3^), then WSA_t_ = 0.8 or 80%. During a disruptive event, if the actual water volumes received dropped to (V_1t_ = 0.04 m^3^, V_2t_ = 0.06 m^3^, V_3t_ = 0.09 m^3^, and V_4t_ = 0.05 m^3^), then WSA_t_ would correspondingly drop to 0.6 or 60%.

In Eq. 11, Pl_t_ is the population impacted at time t, pop_n_ is the population at node n, d_nt_ is a binary variable, q_n_ is the average water volume consumed per day at node n under normal conditions, and R is the average water volume consumed per capita per day. Based on Klise et al.^4^, and industrial experience, R is set to 0.75 m^3^/capita/day and the threshold is 75%.

For the above given example, if q_1_ = q_2_ = q_3_ = q_4_ = 900 m^3^/day, then pop_1_ = pop_2_ = pop_3_ = pop_4_ = 1200 capita. During the given disruptive event, V_1t_/ Ṽ_1t_ = 0.4, V_2t_/ Ṽ_2t_ = 0.6, V_3t_/ Ṽ_3t_ = 0.9, V_4t_/ Ṽ_4t_ = 0.5, and d_1t_ = 1, d_2t_ = 1, d_3t_ = 0, d_4t_ = 1. As a result, the total population impacted at time t, Pl_t_ would equal to 1200 + 1200 + 1200 = 3600 capita.

## Case study: M-City

### WDN and earthquake scenarios

This case study investigates WDN resilience in M-City in Ontario, Canada. The WDN consists mainly of one water treatment plant (WTP) with 125,000 m^3^/d capacity, 7 low-lift pumps and 5 booster pumps, nearly 8000 pipes and 9000 junctions, 1700 valves, one reservoir, and 6 storage tanks. Pipes are made of cast iron, ductile cast iron, and polyvinyl chloride, and no indication for soil liquefaction potential in the serviced area. The spatial layouts of these characteristics were used to determine pipe fragility curves. This system serves more than 65,000 consumers in which 80% of water outflow is consumed by commercial greenhouses.

**Figure 2:**
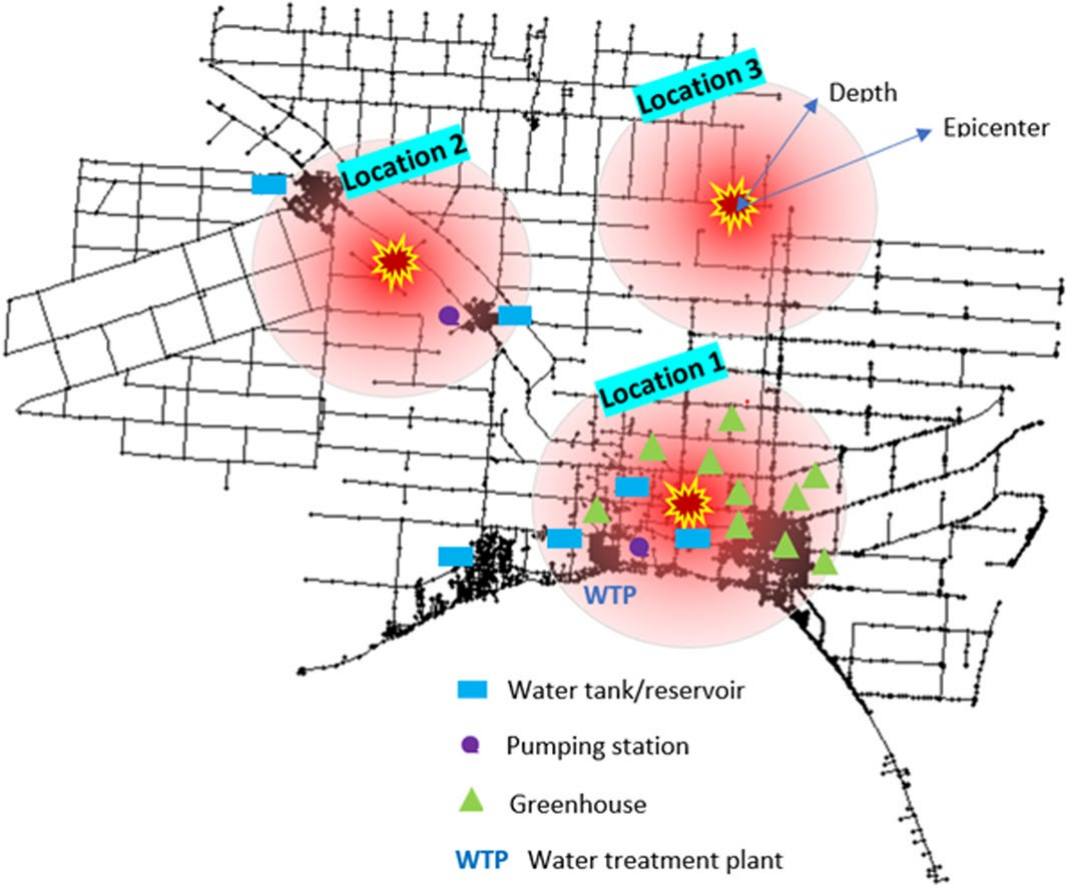
Studied WDN layout and earthquake scenarios configuration.

As for earthquake scenarios, a total of 27 different configurations were selected. These configurations were the outcome of 3 locations (i.e., Location 1, Location 2, and Location 3) within the WDN, 3 magnitudes (i.e., 4.5 M, 5.5 M, and 6.5 M), and 3 possible depths (i.e., 5 km, 10 km, and 15 km). As mentioned before, 50 realizations for each of the 27 scenarios were run in consideration of simulation uncertainty. Figure 2 shows the layout of the WDN and a configuration of the simulated earthquake locations. Location 1 was selected to have an earthquake epicenter close to crucial assets such as the network WTP, multiple storage tanks, pumping station, and strategic consumers (the greenhouses). Location 2 has a medium spatial profile where the earthquake epicentre was selected close to a residential area, booster pumps, and a storage tank. Following this a less dense residential area was chosen as the epicentre for earthquake at Location 3.

All earthquake scenarios are assumed to have occurred 2 days after the simulation has started and continued for 2 weeks. These settings were set to allow adequate simulation before and after the earthquake, and can vary depending on the anticipated damage-repair time frame. A minimum pressure of zero and a nominal pressure of 25 psi were used for the PDD hydraulic simulation runs^4^. All simulated scenarios included possible damages to WDN components, and assumed repair crews were on site 3 h after the shaking occurred.

### WDN damage and repair

**Figure 3:**
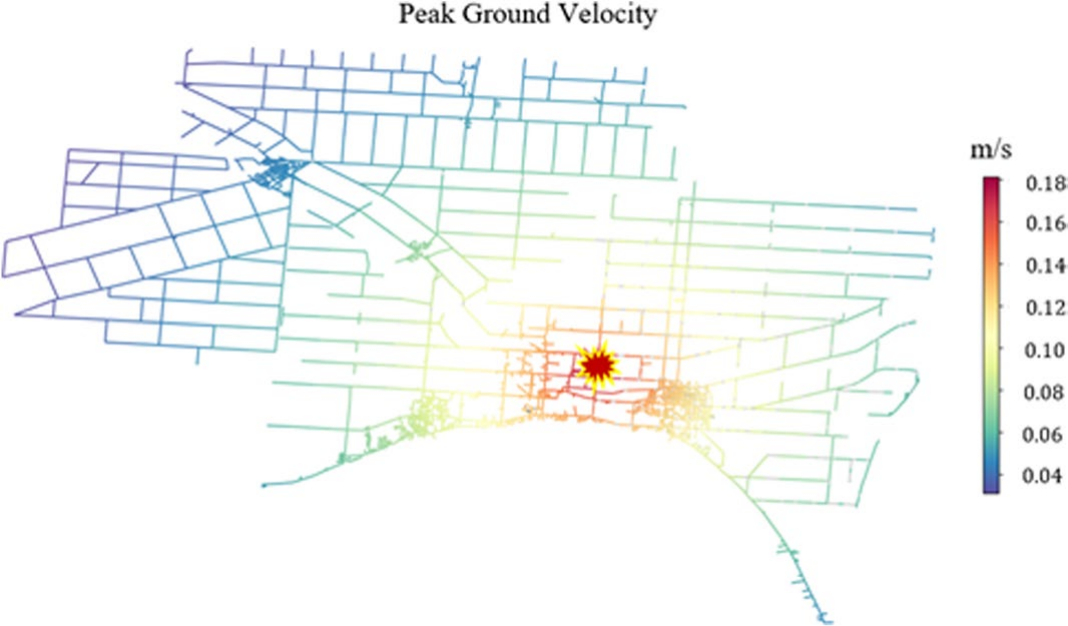
Spatial representation of computed PGV for a 5.5 M, and 15 km depth earthquake at Location 1.

Damages to WDN components were determined after computing PGA, PGV, and RR for each realization of each scenario. Table 4 displays the computation summary of these characteristics and the number of damaged components. For example, the spatial representation of the computed PGV for a 5.5 M, and 15 km depth earthquake at Location 1 is shown in Fig. 3. PGV is at its highest at the epicentre of the earthquake with a value of 0.18 m/s. This value decreases as PGV is computed further away from the earthquake epicentre to reach a value of zero eventually.

**Table 4:** Computation of GMPEs and the number of damaged WDN components.

Location	Location 1			Location 2			Location 3		
Magnitude	4.5 M	5.5 M	6.5 M	4.5 M	5.5 M	6.5 M	4.5 M	5.5 M	6.5 M
**PGA (m/s**^**2**^**)**									
Min	0.201	0.482	1.275	0.192	0.464	1.085	0.197	0.478	1.228
Avg	0.495	1.127	3.138	0.345	0.796	1.863	0.204	0.775	2.236
Max	0.684	1.645	4.483	0.675	1.624	4.768	0.689	1.610	4.747
**PGV (m/s)**									
Min	0.011	0.031	0.173	0.009	0.021	0.144	0.011	0.033	0.157
Avg	0.025	0.102	0.575	0.011	0.054	0.307	0.012	0.064	0.339
Max	0.033	0.181	0.991	0.032	0.183	0.991	0.031	0.184	0.977
**RR (repair/km)**									
Min	0.010	0.017	0.056	0.008	0.014	0.044	0.006	0.013	0.041
Avg	0.018	0.031	0.147	0.011	0.028	0.098	0.008	0.023	0.096
Max	0.024	0.061	0.283	0.014	0.052	0.248	0.10	0.046	0.236
**Number of damaged pipes**	23	91	471	14	83	411	14	77	382
**Number of damaged tanks**	0	2	3	0	1	2	0	0	1
**Number of damaged pumps**	0	4	9	0	2	6	0	0	2

WDN damaged components were determined through predefined damage states (DS) and fragility curves. In this study, pumps, tanks, and pipes were the WDN components that were investigated under seismic hazard. Pumps are either not damaged or shut off (DS1) caused by power outage or machinery failure. For tanks, three damage states were predefined. DS1, DS2, and no damage, representing a minor leak (leak diameter is less than 0.25 m), a major leak (leak diameter is more than 0.25 m with an upper bound of 1.0 m), and no leak, respectively. Also, three damage states were predefined for pipes. DS1 for a minor leak for which the leak diameter was drawn from a uniform distribution with a minimum of 0.01 m and a maximum of 0.05 m, while DS2 represented a major leak with a diameter from 0.05 m to 0.15 m^4^, and DS3 for no damage.

**Figure 4:**
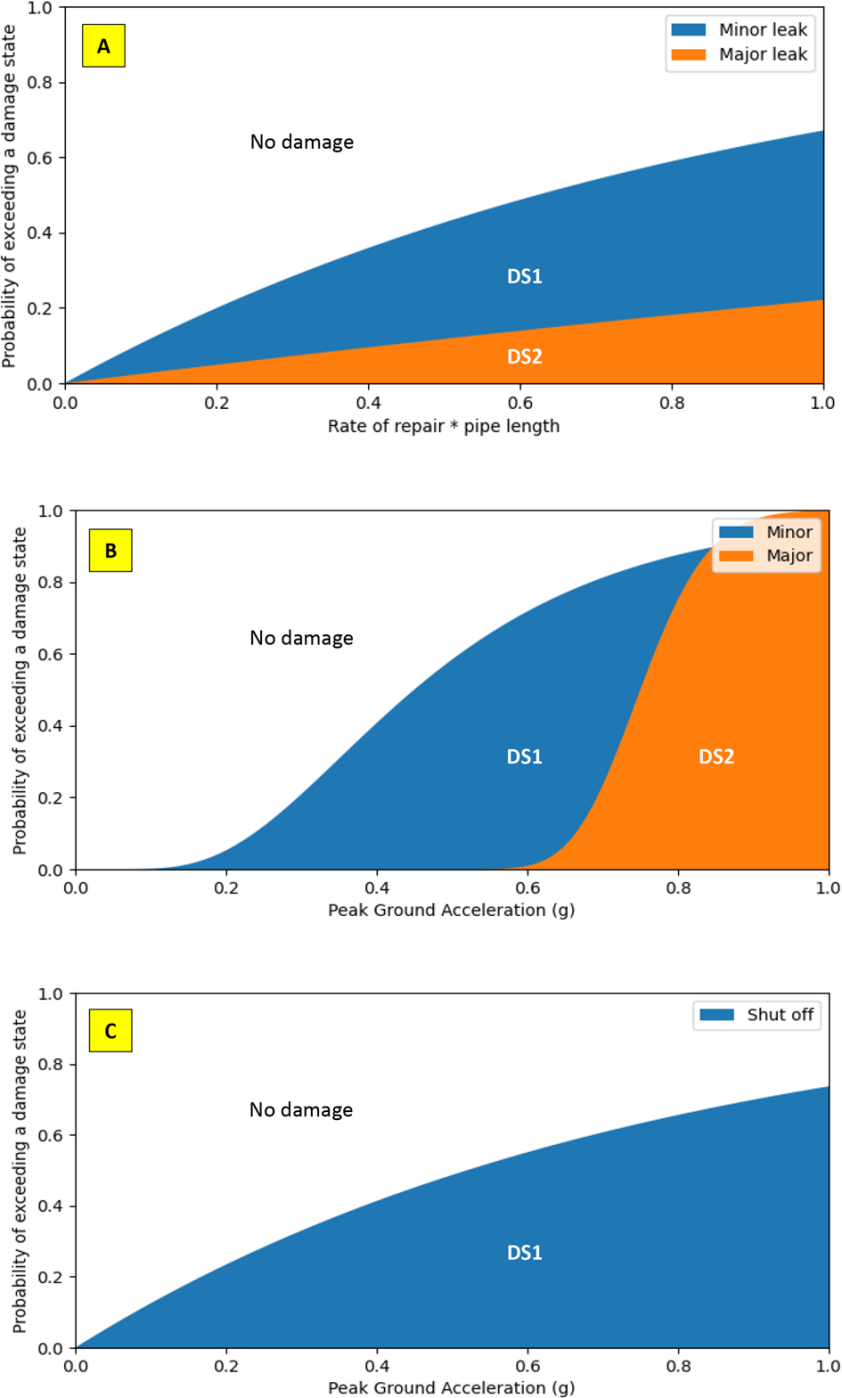
Fragility curves and damage states for (**A**) pipe, (**B**) tank, and (**C**) pump.

All WDN components were assigned a damage state status stochastically based on the probability of exceeding a damage state and the computed PGA, and RR of that specific scenario and realization (i.e., from components’ fragility curve). Figure 4 depicts fragility curves for pipes, tanks, and pumps, and shows the assigned damage states for each. For example, in Fig. 4A if the computed value of RR multiplied by pipe length is equal to 0.4 and has a probability of exceeding a damage state equal to 0.2, then that pipe will most likely sustain a minor leak damage and DS1 is assigned here.

After assigning each component a damage state, the WDN model is updated to include such changes at the time of failure. For example, an “off” control is added to represent the pump damage at the time of damage during a hydraulic simulation. In the same fashion, external nodes are added to represent a leak in pipes or tanks where the node acts as an emitter with a diameter equal to leak diameter.

As WDNs are critical infrastructure and represent community lifelines, repairing resulted damages is imperative. Therefore, two repair strategies (RS) were defined and simulated for all realizations. Paez et al.^31^ presented multiple criteria to consider when repairs are simulated. Two main aspects were adopted from Paez et al.^31^ in this work: (1) large leaks and breaks were prioritized over smaller ones, and (2) crews spend some time locating and isolating leaks before repairs start. The first strategy in this paper, RS1, assumes that customer expected demand was shrunk by one third for 10 days after the earthquake. While the customer expected demand was reduced by half for the second repair strategy, RS2. For both repair strategies, ten crews were deployed to fix damages 4 h after the earthquake. The ten crews include one crew to fix pump damage, two crews to fix tank damage, and seven crews to fix pipe damage. The repair mechanism was adopted from Klise et al.^4^. Where, the pump repair crew fixed one pump every 8 h. Pumps near the treatment plant and reservoir were prioritized as they are at the lowest point of the WDN near the coast. Repaired pumps were switched back on one pump every 8 h.

Tank and pipe crews spent 6 h to find and isolate the largest leaks through closing valves at the nearest junction, and 6 h to fix the leak and open the valve. Considering the number of deployed crews, the one tank and 7 pipes with the largest cumulative leak demand were prioritized for repair. As pressure fluctuates with time, the cumulative leak demand does as well. Therefore, this process of quantifying the cumulative leak demand and prioritizing repair was repeated every 12 h. It is here assumed that once damaged components are repaired, they are brought back to full operating capacity.

## Results and discussion

### Pressure

**Figure 5:**
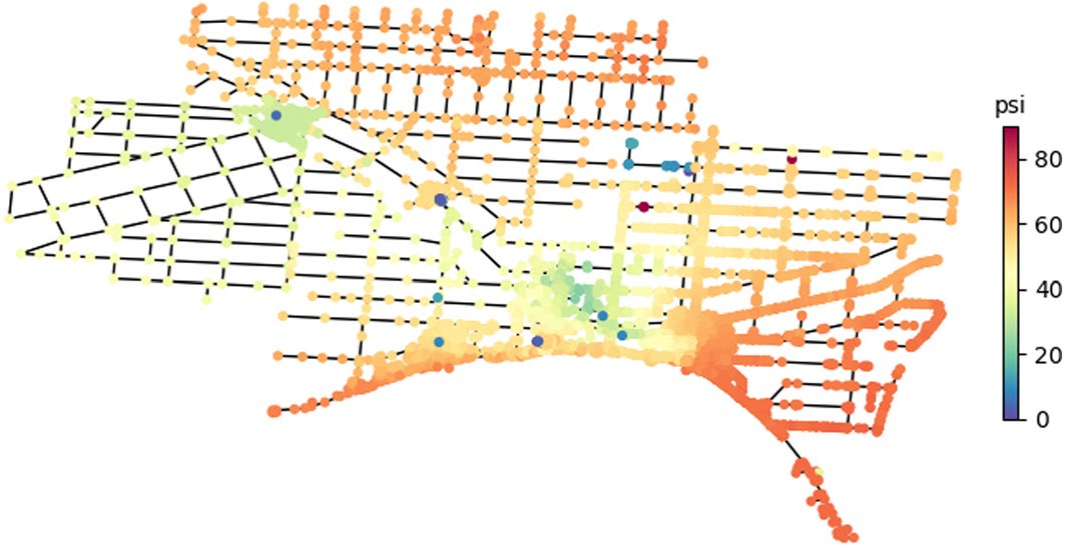
WDN pressure before the occurrence of earthquake and 48 h after the simulation started.

**Figure 6:**
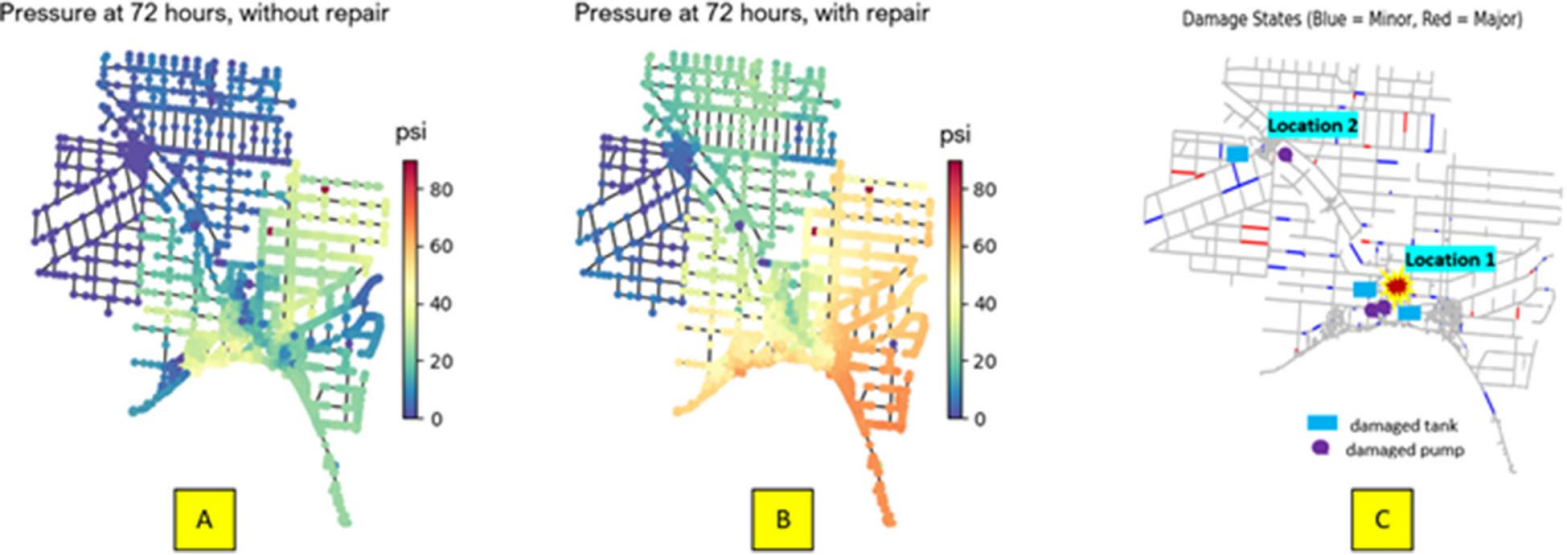
M-City WDN under a 5.5 M earthquake at Location 1. (**A**) Network junction pressure 24 h after the earthquake in the case of no repairs. (**B**) Network junction pressure 24 h after the earthquake in the case of repair strategy, RS2. (**C**) Network damaged components for this scenario.

Tank and junction pressure decline as more WDN components sustain damage. Figure 5 shows the WDN before the occurrence of earthquake. The illustration is at 48 h of the simulation where the pressure ranges mostly between 35 to 80 psi. Figure 6 displays junction pressure 24 h after the earthquake (i.e., at day 3 of the simulation) for a single realization of 5.5 M at Location 1 with and without RS2. For this scenario, 2 pumps near the treatment plant and 2 booster pumps were damaged (e.g., shutoff state) along with 3 tank leaks and 91 pipe leaks. In Fig. 6A, the simulation for this scenario is run without any repair strategy. It can be seen that 24 h after the earthquake, the majority of the network junctions had a pressure below or equal to the nominal pressure of 25 psi. In Fig. 6B, repair crews were sent out and RS2 was implemented. Junction pressures near Location 1 and other parts of the network were starting to increase to 25 psi to 60 psi, whereas insignificant changes are noticed near Location 2. This is, again, due to priority given to fix pumps, tanks, and components near the water treatment plant. And, because a major damage state occurred at multiple pipes near Location 2, as Fig. 6C shows.

Another point to ponder is that junction pressure for this scenario with RS2 was restored to equal or above nominal pressure within a day. This is considered a relatively quick restoration and likely a result of the assumptions of repair strategy RS2 where the customer expected demand dropped by half. This level of drop in customer expected demand might be unrealistic and extreme. However, it mimics an emergency state which is very likely to happen in an earthquake scenario.

**Figure 7:**
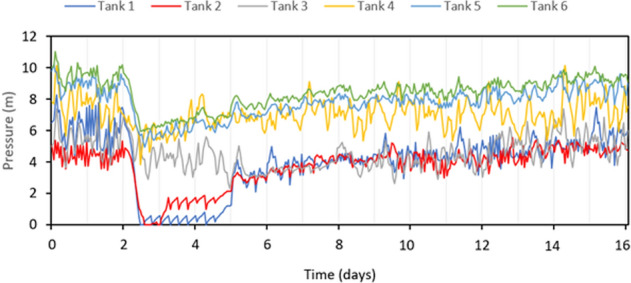
M-City WDN tank pressure under a 5.5 M earthquake at Location 1, and repair strategy, RS2.

Tanks provide storage, supply firefighting demand, and additional head to the water network to meet pressure thresholds. During a seismic event, tank level measured as pressure head (m) is expected to drop as a result of pump damage, and leaks. Figure 7 presents tanks pressure for the single realization mentioned above. For this realization, 3 tanks are affected by direct damage (Tank 1, and Tank 2 at Location 1, and Tank 3 at Location 2). As Fig. 7 shows, the pressure in these three tanks dropped drastically about 4 to 6 h after the earthquake. This is due to shutoff damage for two pumps at Location 1 and one pump at Location 2, and new major and minor leaks in pipes and tanks. This major drop in pressure drains not only the directly affected tanks but also other tanks in the system (i.e., Tanks 3, 4, and 5). As repairs take place to control leaks and bring the damaged pumps back to the system, tank pressure is recovered slowly after about 48 h for Tanks 1, 2, and 3, and on a faster pace for Tanks 4, 5, and 6. Again, tank pressure recovery time depends on the intensity and damage of the earthquake, and repair strategy. So, a longer recovery time is expected for: (1) a higher magnitude earthquake (i.e., 6.5 M earthquake scenarios in our study), and (2) repair strategies with less crews or higher customer expected demand, as in RS1.

### Leak demand

**Figure 8:**
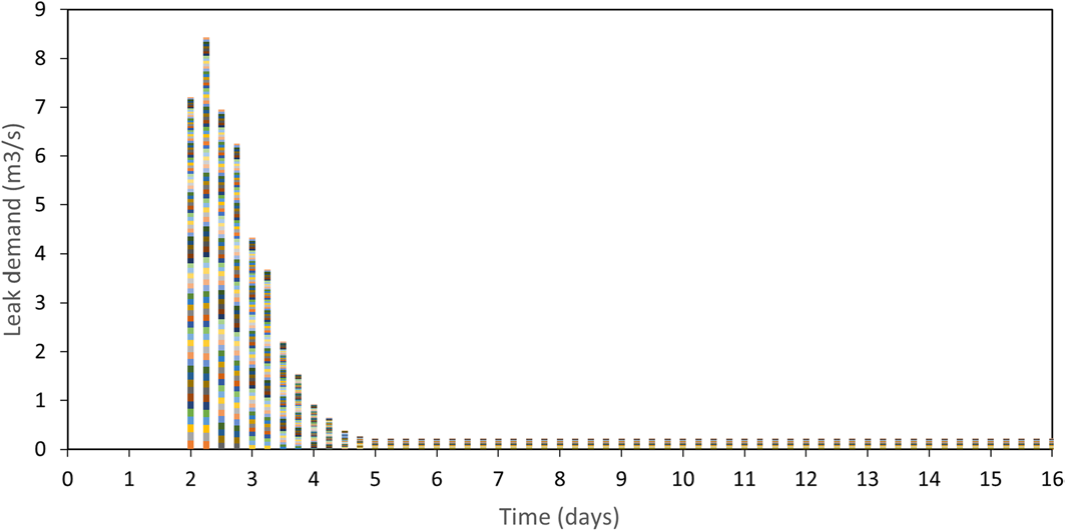
A stacked column chart of leak demand (m3/s) for damaged WDN components caused by a 5.5 M earthquake at Location 1 with RS2. Each coloured block in the stacked column represents a WDN component.

Leak demand associated with minor and major leaks and breakages in pipes and tanks is shown in Fig. 8. The 5.5 M earthquake occurred at Location 1 at day 2 of the simulation and caused leak damage in 91 pipes and 3 tanks. The shown stacked leak demand starts at a high rate during the first 6 h. With RS2 implemented and repair crews detecting, isolating, and fixing these leaks, leak demand drops to half with the first day after the earthquake. Prioritizing higher leak demand rate pipes helps reduce leaks quickly. This can be seen between day 2 and day 4. Between day 2 and day 3, the cumulative leak demand drops by 50% from 8.5 m^3^/s to 4.2 m^3^/s. This compares to 75% from 4.2 m^3^/s to 0.9 m^3^/s for the period between day 3 and day 4. Detecting such leaks above or under ground in a spatial network is a non-trivial task. This highlights the importance of investing in faster and more accurate leak detecting techniques.

It is worth mentioning that pipes that had a high probability of major damage in one earthquake scenario still had a high probability of major or minor damage in another earthquake scenario. A list of these pipes was made, and recommendations were passed to the water utility in order to prioritize retrofitting or replacing them with seismic-resistant pipes.

### Water serviceability

**Figure 9:**
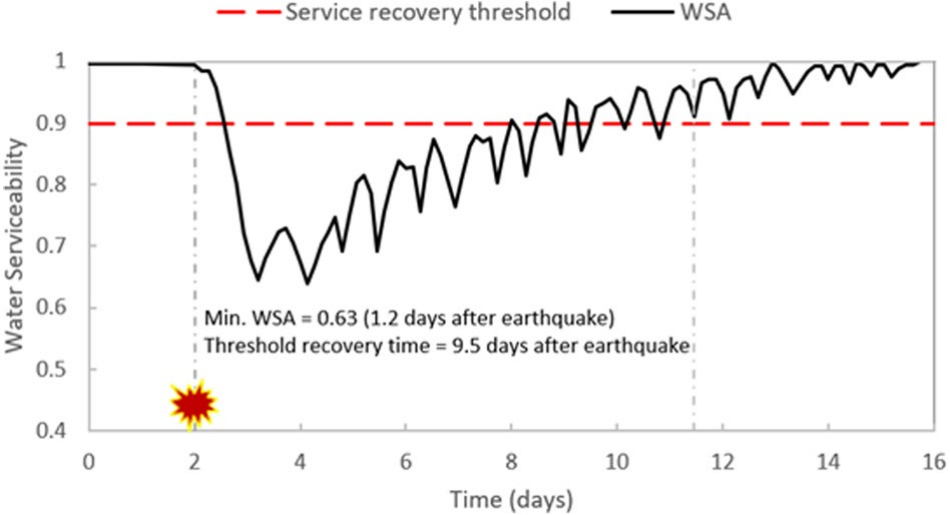
WDN water serviceability represented by the median of 50 realization for the scenario of 5.5 M earthquake at Location 1 with repair strategy RS2.

WDN water serviceability (WSA) is a hydraulic resilience metric that measures the time required for the system to restore its operability. While recovering full serviceability (i.e., serviceability prior to earthquake) is desirable, it might not be attainable within a short time frame. Therefore, a service recovery threshold is set to equal 90% of pre-earthquake WSA. Figure 9 presents the median line (in black) of 50 realizations for the 5.5 M earthquake scenario at Location 1 with repair strategy RS2. It is discernible that as soon as the earthquake occurred at day 2 of the simulation, WSA began to drop down and reached a minimum of 63%, 1.2 days after the earthquake. Repair crews in RS2 started recovering some components 12 h after the earthquake. And as repairs and recovery continued, WSA increased to 74% at 1.8 days after the earthquake. Then, WSA took the shape of a nonuniform signal with increasing highs and lows. This behaviour represents the relationship between actual delivered water volume and customer expected demand (i.e., the definition of WSA). This nonuniform signal shape depicts the varying customer expected demand during the day and from one day to another (weekday versus weekend patterns). About 9.5 days after the earthquake, the WDN restored a minimum of 90% of its water serviceability while it took about 2 weeks to go fully back to a normal state with continuous repairs.

Table 5 summarizes minimum WSA and recovery time for all scenarios studied in this paper. It can be seen that minimum WSA is lower, and recovery time is higher for: (1) earthquakes with higher magnitude that caused more damage, (2) earthquakes that occurred at Location 1, then those at Location 2, and last at Location 3. This is explained by the density of strategic and valuable assets’ location within the WDN, and (3) repair strategy that had a lower reduction in customer expected demand (i.e., RS1 with a reduction of one third compared to half for RS2).

**Table 5:** Minimum water service availability (WSA) and recovery time for studied earthquake scenarios. Values are computed using the median of the 50 realizations for each scenario.

Repair strategy	Magnitude	Location 1		Location 2		Location 3	
		Min. WSA	Recovery time (days)	Min. WSA	Recovery time (days)	Min. WSA	Recovery time (days)
RS1	4.5	0.69	5.2	0.87	1.4	0.96	0.0^a^
	5.5	0.59	10.1	0.67	8.4	0.77	3.6
	6.5	0.41	12.9	0.49	11.9	0.61	8.8
RS2	4.5	0.76	3.8	0.91	0.0	0.93	0.0
	5.5	0.63	9.5	0.65	9.1	0.74	4.1
	6.5	0.47	12.3	0.54	10.6	0.64	8.1

^a^A recovery time of 0 implies that the median value for WSA never dropped below 90% water service availability.

### Population impacted

**Figure 10:**
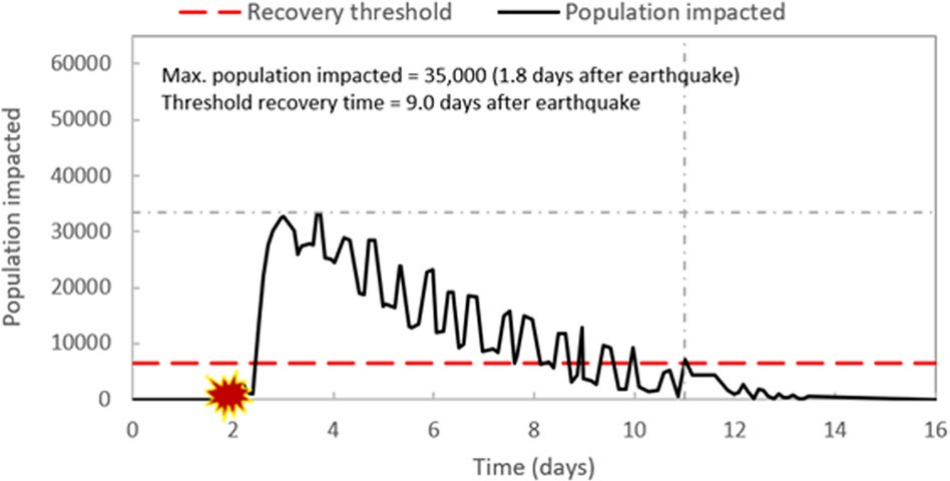
Population impacted represented by the median of 50 realization for the scenario of 5.5 M earthquake at Location 1 with repair strategy RS2.

The last resilience metric presented in this paper is quantifying the number of people affected by disruptions due to seismic hazard. Figure 10 shows the population impacted represented by the median of 50 realizations for the scenario of the 5.5 M earthquake at Location 1 with repair strategy RS2. One day after the earthquake, water service for about 33,000 people was impacted by the earthquake, as Fig. 10 shows. This number reached its maximum of 33,500 people 1.8 days after the earthquake. As repairs took effect 12 h after the earthquake, more components were brought back to service. The number of people impacted started to drop after 2 days and this drop took a nonuniform signal shape as mentioned before. Nine days after the earthquake, the number of people impacted reached the assumed recovery threshold of 6500 people (i.e., 10% of the total population).

Table 6 summarizes the maximum population impacted and recovery time for all scenarios studied in this paper. Similar to WSA, the maximum population impacted is higher, and recovery time is higher for: (1) earthquakes with higher magnitude that caused more damage, (2) earthquakes that occurred at Location 1, then those at Location 2, and last at Location 3, and (3) repair strategy that had a lower reduction in customer expected demand (i.e., RS1 with a reduction of one third compared to half for RS2).

**Table 6:** Maximum population impacted and recovery time for studied earthquake scenarios. Values are computed using the median of the 50 realizations for each scenario.

Repair strategy	Magnitude	Location 1		Location 2		Location 3	
		Max. population impacted	Recovery time (days)	Max. population impacted	Recovery time (days)	Max. population impacted	Recovery time (days)
RS1	4.5	19,642	6.3	14,632	0.8	4952	0.0^a^
	5.5	37,765	10.6	23,854	6.9	15,901	0.8
	6.5	56,931	13.8	48,525	11.1	29,746	7.6
RS2	4.5	17,534	5.6	11,490	0.5	1632	0.0
	5.5	33,500	9.0	20,752	6.4	8217	0.5
	6.5	52,395	11.7	43,851	10.8	22,864	6.8

^a^A recovery time of 0 implies that the median value for population impacted never dropped below 10% of total population.

## Conclusions

An earthquake generation model and a probabilistic flow-based PDD hydraulic model were coupled and applied to an actual WDN in M-City Ontario, Canada. A review of the relevant literature revealed that very few studies applied PDD analysis when simulating a simultaneously disruptive earthquake, or when PDD was adopted, probabilistic simulations were not considered. Further, those simulations that applied DDA assumed that the nodal demand was met regardless of pressure gradient (atypical during a disruptive event like an earthquake). Other studies applied PDD without considering the wide range of possible simulations (i.e., probabilistic simulation), or they were applied on an imaginary WDN.

This study included earthquake scenarios with three different magnitudes and three different depths at three locations within the WDN. These scenarios were simulated along with two repair strategies to measure the WDN resilience. WDN components were damaged under seismic hazard. The damage was defined by the probability distribution functions in the fragility curves for each component. Hydraulic metrics were measured to quantify the WDN resilience.

The following major summary remarks can be made based on our study:

- Light earthquakes (represented by the 4.5 M scenarios) tended to cause minimal damage to WDNs, damage typically occurred in poorly maintained and aged components. This damage became significant in moderate earthquake scenarios (represented by the 5.5 M scenarios), and severe in strong earthquake scenarios (represented by the 6.5 M scenarios).
- Water service could be lost for days and up to 90% of water serviceability and population could be impacted in moderate and strong earthquake scenarios.
- Repair strategies significantly affect the time of restoration and should be planned and updated in a utility’s WSP periodically.
- Aged infrastructure components (e.g., old pipes) should be replaced by seismic-resistant material, particularly in sensitive and densely populated locations (i.e., Location 1 in our study).

Water utilities now have an expanding sphere of threats to prepare for that include aging infrastructure, natural disasters, and man-made hazards. This work illustrates a useful approach to help more water utilities assess their critical infrastructure under potentially significant seismic scenarios. The tools illustrated herein can be engaged to extend or enhance essential utility water safety plans. That said, the findings in this work required large input datasets, lengthy analysis, and a set of assumptions. For similar analysis and for a real-world WDN like the one studied in this manuscript, a well calibrated model of the system and earthquake model data should be available and fed to the sub-modules presented previously in Fig. 1. During this work, some of the input datasets were not available and had to be generated. This emphasizes on the importance of data and the challenges of data scarcity for water utilities.

Some assumptions were made in this study about the repair strategy and the number of crews. These assumptions do not represent all water utilities but rather this case study. This should not affect the validity and importance of such analysis, however, should be taken in consideration for other locations. Future work will investigate the possible occurrence of fire and contamination events and during and earthquake. Similar thorough analysis shall be applied and further improved.

## Data Availability

The data that support the findings of this study are available from the Union Water Supply Systems, but restrictions apply to the availability of these data, which were used under license for the current study, and so are not publicly available. Data are however available from the authors upon reasonable request and with permission of the Union Water Supply Systems.
